# Treatment Motivations and Expectations in Patients with Actinic Keratosis: A German-Wide Multicenter, Cross-Sectional Trial

**DOI:** 10.3390/jcm9051438

**Published:** 2020-05-12

**Authors:** Theresa Steeb, Anja Wessely, Dagmar von Bubnoff, Thomas Dirschka, Konstantin Drexler, Conrad Falkenberg, Jessica C. Hassel, Kinan Hayani, Svea Hüning, Katharina C. Kähler, Sigrid Karrer, Christian Krammer, Ulrike Leiter, Diana Lill, Enklajd Marsela, Andreas Meiwes, Dorothée Nashan, Suzan Nasifoglu, Lutz Schmitz, Judith Sirokay, Alexander Thiem, Jochen Utikal, Alexander Zink, Carola Berking, Markus V. Heppt

**Affiliations:** 1 Department of Dermatology, Universitätsklinikum Erlangen, Friedrich-Alexander-University of Erlangen-Nürnberg (FAU), 91054 Erlangen, Germany; Theresa.Steeb@uk-erlangen.de (T.S.); Anja.Wessely@uk-erlangen.de (A.W.); Carola.Berking@uk-erlangen.de (C.B.); 2 Department of Dermatology and Allergy, University Hospital, LMU Munich, 80337 Munich, Germany; Kinan.Hayani@med.uni-muenchen.de (K.H.); christiankrammer@gmx.de (C.K.); Diana.Lill@med.uni-muenchen.de (D.L.); Enklajd.Marsela@med.uni-muenchen.de (E.M.); Suzan.Nasifoglu@med.uni-muenchen.de (S.N.); 3 Comprehensive Cancer Center Erlangen-European Metropolitan Area of Nuremberg (CCC ER-EMN), 91054 Erlangen, Germany; 4 Department of Dermatology, University Hospital Schleswig-Holstein Campus Lübeck, 23562 Lübeck, Germany; dagmar.bubnoff@uniklinik-freiburg.de; 5 Centroderm Clinic, 42287 Wuppertal, Germany; t.dirschka@centroderm.de (T.D.); Conrad.Falkenberg@med.uni-duesseldorf.de (C.F.); 6 Department of Dermatology, University Hospital Regensburg, 93042 Regensburg, Germany; Konstantin.Drexler@klinik.uni-regensburg.de (K.D.); Sigrid.Karrer@klinik.uni-regensburg.de (S.K.); 7 Section of Dermatooncology, Department of Dermatology and National Center for Tumor Diseases, 69120 Heidelberg, Germany; Jessica.Hassel@med.uni-heidelberg.de; 8 Department of Dermatology, Hospital of Dortmund, 44137 Dortmund, Germany; Svea.Huening@klinikumdo.de (S.H.); Dorothee.Nashan@klinikumdo.de (D.N.); 9 Department of Dermatology, University Hospital Schleswig-Holstein Campus Kiel, 24105 Kiel, Germany; kkaehler@dermatology.uni-kiel.de; 10 Department of Dermatology, Center for Dermatooncology, University Hospital Tübingen, 72076 Tübingen, Germany; Ulrike.Leiter@med.uni-tuebingen.de (U.L.); Andreas.Meiwes@med.uni-tuebingen.de (A.M.); 11 Department of Dermatology, Skin Cancer Center, Ruhr University Bochum, 44801 Bochum, Germany; luschmitz@hotmail.de; 12 Department of Dermatology and Allergy, University Hospital of Bonn, 53127 Bonn, Germany; Judith.Sirokay@ukbonn.de; 13 Clinic for Dermatology and Venereology, University Medical Center Rostock, 18057 Rostock, Germany; Alexander.Thiem@med.uni-rostock.de; 14 Skin Cancer Unit, German Cancer Research Center (DKFZ) and Department of Dermatology, Venereology and Allergology, University Medical Center Mannheim, Ruprecht-Karl University of Heidelberg, 68167 Mannheim, Germany; Jochen.Utikal@umm.de; 15 Department of Dermatology and Allergy, Technical University of Munich, 80802 Munich, Germany; alexander.zink@tum.de

**Keywords:** skin cancer, patient education, actinic keratosis, cross-sectional study, patient-centered care, patient-reported outcomes, personalized medicine

## Abstract

Patient-centered motives and expectations of the treatment of actinic keratoses (AK) have received little attention until now. Hence, we aimed to profile and cluster treatment motivations and expectations among patients with AK in a nationwide multicenter, cross-sectional study including patients from 14 German skin cancer centers. Patients were asked to complete a self-administered questionnaire. Treatment motives and expectations towards AK management were measured on a visual analogue scale from 1–10. Specific patient profiles were investigated with subgroup and correlation analysis. Overall, 403 patients were included. The highest motivation values were obtained for the items “avoid transition to invasive squamous cell carcinoma” (mean ± standard deviation; 8.98 ± 1.46), “AK are considered precancerous lesions” (8.72 ± 1.34) and “treating physician recommends treatment” (8.10 ± 2.37; *p* < 0.0001). The highest expectation values were observed for the items “effective lesion clearance” (8.36 ± 1.99), “safety” (8.20 ± 2.03) and “treatment-related costs are covered by health insurance” (8.00 ± 2.41; *p* < 0.0001). Patients aged ≥77 years and those with ≥7 lesions were identified at high risk of not undergoing any treatment due to intrinsic and extrinsic motivation deficits. Heat mapping of correlation analysis revealed four clusters with distinct motivation and expectation profiles. This study provides a patient-based heuristic tool for a personalized treatment decision in patients with AK.

## 1. Introduction

Long-term exposure to ultraviolet (UV) radiation can lead to the formation of actinic keratoses (AK) in light-skinned individuals [1,2]. Lesions present as diffuse red and keratotic or scaling plaques with a rough, sandpaper-like surface on chronically sun-exposed areas such as the face, ears, arms, and dorsal hands [2,3]. AK lesions are considered precursors of invasive cutaneous squamous cell carcinoma (cSCC), although the conversion risk for an individual lesion to progress into cSCC is estimated low [4]. The presence of multiple lesions, marked basal proliferation in histology, and additional signs of chronic UV damage on the adjacent skin increases the risk for progression considerably, and spontaneous regression is less likely to occur [5,6,7]. As it is clinically not possible to exactly predict which AK will become invasive cSCC, international treatment guidelines recommend early and consequent treatment [8,9]. Today, numerous interventions with varying efficacy and safety profiles are licensed for the management of AK. These comprise lesion-directed therapies such as excision or cryosurgery as well as field-directed therapies including photodynamic therapy (PDT) or topical interventions, which target a whole area of skin bearing multiple AK and aim at clearing subclinical changes [10].

However, the individual effect of the respective treatment strongly depends on patients’ willingness and consent to adhere to the treatment regimen. Almost all AK treatments carry a therapeutic burden such as pain, adverse events, costs, treatment duration, altered cosmetic appearance, local skin reactions or inconvenience of application. These factors may influence the underlying motives of patients to be willing to undergo or choose a specific intervention [11]. Understanding treatment motivation, expectations, and individual patient preferences critically influence the treatment success. Besides, these factors are important to improve the acceptability of and compliance to treatment regimens [11,12]. Surprisingly, the patient-centered motives and expectations towards AK treatment have received little attention until now but can represent a major barrier for treatment adherence [13,14,15]. Here, we report the results of a German-wide, multicenter, cross-sectional study to gain insight into the management of AK by investigating patient attitudes, expectations, and motives.

## 2. Materials and Methods

### 2.1. Study Design and Ethics Approval

A multicenter, cross-sectional study that included patients from 14 German skin cancer centers was conducted between May and August 2019. This study was approved by the institutional review board of the University Hospital (LMU Munich) on 7 June 2019 (approval number 19-356 KB, Supplementary A1). We closely adhered to the STROBE statement for cross-sectional studies for the reporting of this study (Supplementary A2) [16,17].

### 2.2. Setting and Participants

Adult patients (≥18 years) presenting with AK in the participating centers were asked either by a physician or a nurse to complete a self-administered four-page questionnaire (purposive sampling, Supplementary A3). As the first page of the questionnaire included questions related to previous treatments, number, and localization of AK, patients were allowed to ask the physician for advice and to obtain patient-specific information, if necessary. Participation was voluntary and all participants gave verbal informed consent before completing the questionnaire. Refusals were not documented, and no incentives were provided. Relatives or accompanying persons were excluded from the study. Each patient was allowed to participate only once in the survey (cross-sectional design).

### 2.3. Survey

As no validated survey tools for the objective of our study existed, the questionnaire was developed *de novo* based on a literature review and thorough dermatologic expert consulting. The questionnaire included items on previous treatments for AK, localization and number of lesions, immunosuppression, underlying motives to undergo AK treatment and expectations and wishes towards therapy as well as basic demographic information (age, gender, marital status, health insurance status, profession). Patients with an increased UV exposure due to their long-term profession were categorized as at high risk for developing skin cancer. For the questions related to motives and expectations towards AK treatment, patients were asked to rate the level of agreement on a continuous visual analogue scale (VAS) ranging from 0 (do not agree) to 10 (fully agree). The full questionnaire is available in Supplementary A3. The questionnaire was pre-tested and validated for clarity and comprehension by independent researchers who were not involved in the design of the original questionnaire and volunteering patients without AK. Unclear items were thoroughly discussed and rephrased until a consensus on clarity was reached. Based on this feedback, questions were simplified, the questionnaire was shortened and finally, the questionnaire was revised to its final version. Completed questionnaires were sequentially numbered for data entry purposes but were not linked to any identifying patient information to assure irreversible anonymity.

### 2.4. Data Analysis

We calculated an estimated sample size of at least *n* = 320 required for this explorative study design as suggested by Tabachnik and Fidell by multiplying the number of the questionnaires’ items by factor 10 [18]. Statistical analyses were conducted with SPSS (IBM SPSS Statistics version 24, IBM Corporation, Armonk, NY, USA). Descriptive analyses included means with standard deviations (SD) or medians and interquartile ranges (IQR). Categorical variables were expressed as frequencies and percentages. Subgroup differences between two groups were explored with the student’s *t*-test or Mann-Whitney-U-test. For the comparison of more than two groups, one-factor analysis of variance followed by Scheffé procedure or the Kruskal-Wallis test was used. The relationship between the level of agreement of patient motives and expectations towards AK therapy were examined with Spearman’s correlation. A two-sided *p*-value < 0.05 was considered statistically significant in all cases. Missing values were excluded pairwise. Besides, missing data were addressed by indicating the number of participants considered in each analysis.

## 3. Results

### 3.1. Characteristics of the Study Population

A total of 403 patients were included. The majority was male (73.7%; 294/399) and the median age at the time of the visit was 77 years with a range from 43 to 94 years. 73.5% (291/396) of the patients were married, 15.7% (62/396) were widowed followed by 5.6% who were divorced (22/396) or single/unmarried (5.3%; 21/396). Besides, most patients had statutory health insurance (76.8%, 304/396). 7.9% (30/382) of patients stated to take immunosuppressive medication, the majority (*n* = 17) due to organ transplantation. Of these, 11 patients were renal transplant recipients, one had a transplanted liver and five transplant recipients did not provide information regarding their transplanted organ. The remaining patients stated to have an autoimmune disease (16.7%, *n* = 5) or to take immunosuppressive medications due to other reasons, such as rheumatoid arthritis (*n* = 1) or ankylosing spondylitis (*n* = 1), representing a risk population for the development of AK (Table 1). The majority of patients (91.0%; 303/333) were classified as not having an increased risk for skin cancer. Patients presented predominantly with AK located in the face or scalp (65.6%; 261/398). In contrast, 23.9% (95/398) had AK both on the scalp and facial sites as well as in non-head and non-facial regions. The remaining 10.6% (42/398) showed AK only on the extremities or trunk. Nearly half of the patients had 1–3 AK treated at the time of the visit (47.6%, 167/351), followed by ≥7 AK (29.9%, 105/351) and 4–6 AK (22.5%, 79/351). Overall, 83.6% (331/396) of the patients reported at least one pre-treatment, whereas 15.4% (61/396) did not have any prior treatment and 1% (4/396) could not remember. Nearly half of the patients (46.4%; 153/330) voted to have had their AK treated at least once with diclofenac sodium 3% in hyaluronic acid 2.5% gel, followed by PDT (38.8%; 128/330) and surgical excision (37.3%; 123/330) (Supplementary Figure S1).

### 3.2. Items of Treatment Motivation

**Figure 1:**
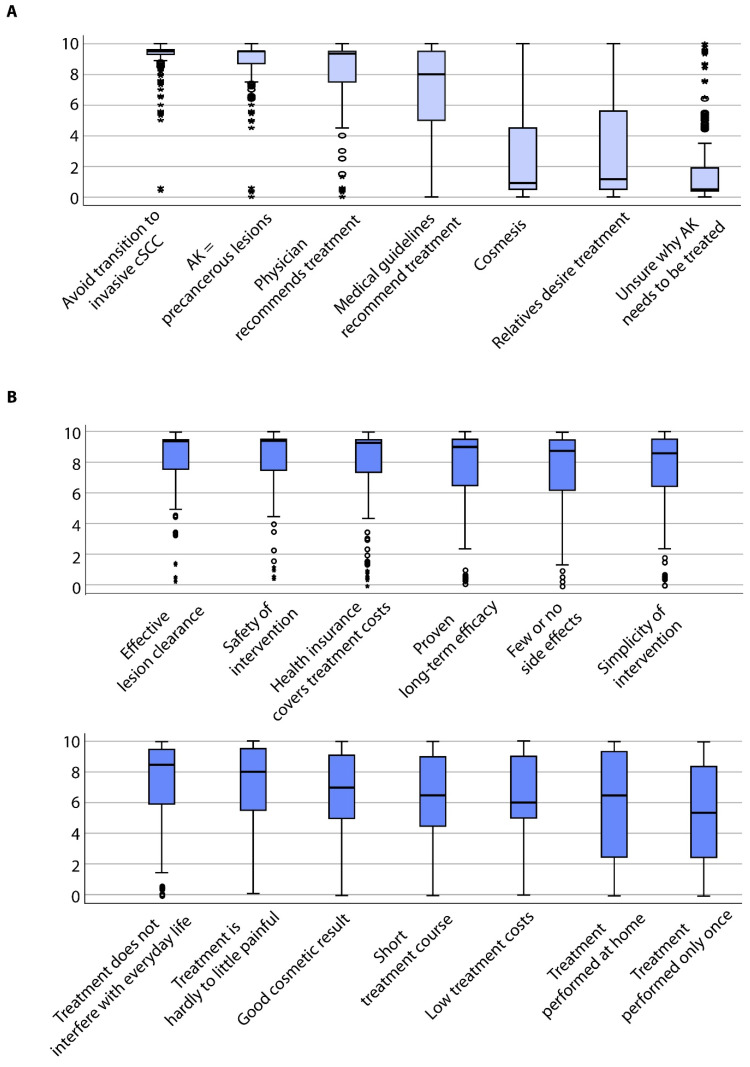
Boxplots showing all patients’ specific evaluation on (**A**) motivation for therapy of their actinic keratoses (AK) and (**B**) on expectations towards therapy of their AK.

**Figure 2:**
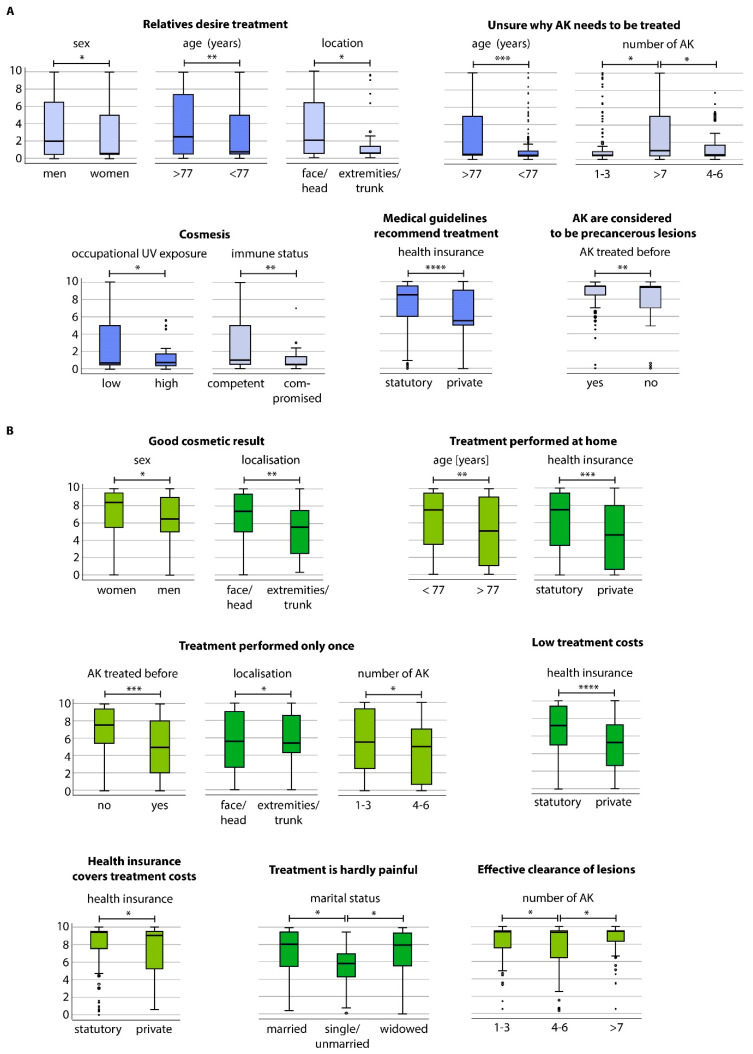
Boxplots showing patients’ specific subgroup evaluation on (**A**) motivation for a therapy of their AK and (**B**) on expectations for the therapy of AK; *p*-values: * <0.05; ** <0.01; *** =0.001; **** =0.000.

Patients strongly agreed to undergo treatment to avoid the transition of AK to invasive cSCC (mean ± standard deviation: 8.98 ± 1.46) or since AK are considered precancerous lesions (8.72 ± 1.34) (Figure 1A). Interestingly, patients also agreed to undergo treatment due to the physician’s recommendation (8.10 ± 2.37) or because medical guidelines recommend treatment (7.19 ± 2.67). In contrast, patients rather disagreed that cosmetic reasons (2.49 ± 2.84) and treatment due to the desire of third parties such as relatives (3.35 ± 3.45) were motivating factors (*p* < 0.0001). Other reasons specifically mentioned by the patients in a free-text field included aesthetic restrictions in general (*n* = 3), improvement of the professional appearance (*n* = 1), pain relief (*n* = 3), or improvement of quality of life (*n* = 1). Next, we investigated whether the treatment motivations varied according to clinical and socio-demographic parameters and performed subgroup analyses. Significant differences among the subgroups are shown in Figure 2A. Further information can be obtained from the supplementary results (Appendix A.1).

### 3.3. Items of Treatment Expectation

Patients strongly expected effective AK lesion clearance (8.36 ± 1.99) (Figure 1B). Safety of the individual interventions was also considered important (8.20 ± 2.03), followed by the coverage of treatment-related costs by health insurance funds (8.00 ± 2.41). Furthermore, patients expected that the treatment has a proven long-term efficacy (7.80 ± 2.32) and few or no side effects (7.77 ± 2.33). Further patient preferences included simplicity of the individual intervention (7.70 ± 2.39), no interference with everyday life (7.43 ± 2.55) and that the treatment is hardly to minimally painful (7.25 ± 2.58). Additionally, patients estimated a good cosmetic outcome (6.60 ± 2.77), short treatment course (6.31 ± 2.97) and little costs (6.24 ± 2.83) as important. Lower values were obtained for home-based treatment (5.77 ± 3.56) and a one-time treatment procedure (5.40 ± 3.35; *p* < 0.0001). Further reasons that were specifically addressed by patients in the free-text field included the wish for regular surveillance and better education regarding the dangers and avoidance of sunlight by physicians (*n* = 2), long-term clearance (*n* = 6) or no occurrence or spread of skin cancer (*n* = 6).

Next, we performed subgroup analysis for the motivation items. Significant differences among the subgroups are shown in Figure 2B. In particular, patients aged >77 years and those with >7 lesions were unsure why treatment was indicated. Further information can be obtained from the supplementary results (Appendix A.2).

### 3.4. Correlation Analysis of Motivation and Expectation Items

**Figure 3:**
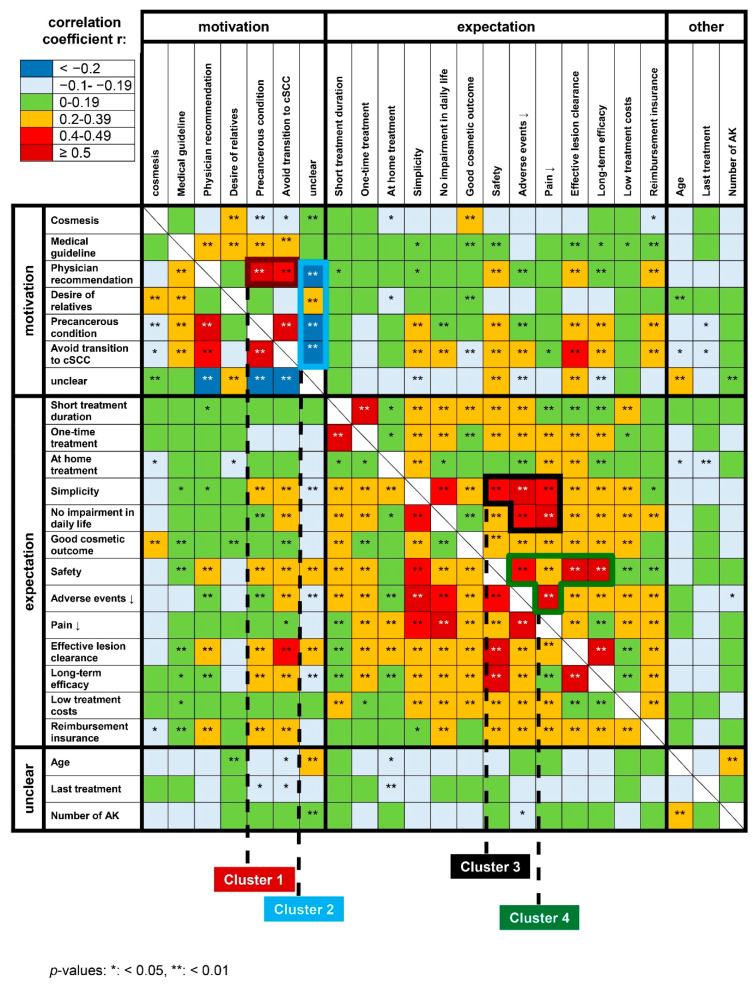
Heat-map showing the correlations of patients’ motives and expectations towards AK management. The correlation coefficients were used to indicate the strength of the correlation. Four patient clusters were identified (black frames).

To identify distinct patient profiles and clusters of patient subgroups, we performed Spearman’s correlation of the individual motivation and expectation items. As most items were correlated with each other, we focused on correlations that were most strongly correlated (i.e., r > 0.5 or r < −0.2). The recommendation of the physician for treatment was positively correlated with the motivation to have AK treated as they are considered precancerous lesions (r = 0.547) (Figure 3). Furthermore, the item “avoid progression to invasive cSCC” was also correlated with the motivation to treat AK because they are perceived precancerous lesions (r = 0.670). There was also a trend that patients who agreed to desire a short treatment wished for treatment to be performed only once (r = 0.557). Patients voting a proven long-term effect as important tended to rate effective lesion clearance to be important (r = 0.682) as well as safe treatment (r = 0.639). Effective lesion clearance was additionally positively correlated with safe treatment (r = 0.535). Another correlation was identified for treatments with hardly or no adverse events and simplicity of treatment (r = 0.606). There was also a correlation between the desire for an intervention that does not interfere with daily life and one that is perceived to be hardly or not painful (r = 0.557). Additionally, the item “unclear why AK needs to be treated” and age were slightly significantly correlated with each other. All correlations were statistically highly significant (*p* < 0.01).

### 3.5. Clustering Treatment Motivation and Expectation Items to Define Distinct Patient Populations

Based on the heat map of these data, we identified two distinct clusters for treatment motivation (clusters 1 + 2) and treatment expectation (clusters 3 + 4), respectively (Figure 3). Cluster 1 comprised the items “physician’s recommendation”, “AK as precancerous condition”, and “avoid transition to invasive cSCC (Table 2). Cluster 2 was dominated by the negatively correlated items “unclear why AK need to be treated”, “physician’s recommendation”, “AK as precancerous condition”, and “avoid transition to invasive cSCC”. Within this cluster “unclear why AK need to be treated” and “desire of relatives” was positively correlated with each other. Cluster 3 comprised “simplicity”, “safety”, “few adverse events”, “little painfulness” and “no impairment in daily life”, and cluster 4 “safety”, “few adverse events”, “little painfulness”, “effective lesion clearance”, and “long-term efficacy”. All items of clusters 1, 3, and 4 were positively correlated.

## 4. Discussion

This cross-sectional study was designed to investigate individual, patient-centered motives and expectations towards the treatment of AK which have not received much attention until now but should ultimately be considered when a treatment choice is made. Furthermore, we aimed to identify distinct patient profiles that could provide a valuable and heuristic resource to facilitate personalized decision-making in the daily routine. For the first time, we define distinct patient profiles for the treatment of AK based on primary patient-derived data. A previous study defined six profiles based on the experience of an expert panel. However, patients themselves or patient representatives were not involved when the profiles were derived [19]. In analogy to other dermatologic conditions, we believe that it is indispensable to use patient-derived data as a primary source to outline specific profiles and to guide patient-centered treatment [20,21]. Thus, we collected data on the treatment motivation and expectation from a large cohort of 403 patients distributed among 14 major centers for AK care within Germany. Overall, the highest motivation for AK treatment was to avoid the transition to invasive cSCC, because AK are considered precancerous lesions, and because the treating physician recommends treatment. These motivation items also achieved uniformly high values on the VAS in the subgroup analysis. The highest expectation values were obtained for effective lesion clearance, the safety of the intervention, and that the treatment costs are covered by health insurance. We conclude that these factors should be considered and highlighted for any treatment decision.

Nowadays, choosing an appropriate and individualized intervention often largely depends on the knowledge, expertise, and preference of the practitioner as well as reimbursement status and may be insufficiently aligned with the individual desire of the patient [22,23]. In a recent qualitative study among physicians, cryosurgery was cited as the predominant therapy because other forms of therapy were little known or because there was uncertainty about their use [24]. Nevertheless, most dermatologists in this qualitative survey stated that they were aiming for guideline-based therapy, which was also an important motivation for patients in our study. Among all items of motivation in our survey, it achieved the third-highest average values, albeit with a high degree of variability. Interestingly, this motivation was higher for patients with statutory health insurance than for those with private health insurance. This could be possibly explained by the fact that patients with statutory health insurance suspect that therapy in line with the guidelines is also fully reimbursed and that this does not result in any financial losses for them. A concrete counterexample is conventional PDT, which is uniformly recommended in current treatment guidelines [8,9] but has not yet been reimbursed by the statutory health insurance funds in Germany.

To further dissect and identify specific profiles, we performed subgroup analyses and correlated the motivation and expectation items with each other and with the baseline characteristics. Cosmesis showed rather low motivation values in the overall population. However, it was rated significantly higher in patients with low occupational UV exposure and a non-immunocompromised status. Furthermore, the expectation of a good cosmetic outcome was higher in women and patients with AK located on the face or head. Interestingly, patients whose AK had never been treated before and those with few AK (1–3 lesions) rather expected a one-time treatment, indicating that they may not yet be aware that AK is a chronic condition, which usually requires multiple treatment modalities and lifelong surveillance [5]. We propose that it is critical to provide substantial information framing on the disease course and to ensure sufficient communication and patient education for this subgroup [25]. Treating physicians must actively approach and educate this subgroup, especially those who undergo AK treatment for the first time.

The correlation analysis between motives and expectations revealed that patients who underwent treatment to prevent progression to invasive cSCC mainly desired an effective, long-lasting, safe, and simple approach that does not interfere with daily life and whose costs are covered by health insurance. Similarly, patients undergoing AK treatment due to the physician’s recommendation preferred an effective and safe therapy that is reimbursed by health insurance. In contrast, those who underwent treatment for cosmetic reasons expected a good cosmetic result while being less interested in the efficacy and safety of the procedure. The heat-map of the correlation analyses revealed four clusters with highly positively (clusters 1, 3, 4) and negatively (cluster 2) correlated items. For motivation, we identified two distinct clusters. While cluster 1 appeared easily and highly motivated for treatment both intrinsically and extrinsically by the physician’s recommendation, cluster 2 may have a high risk of not undergoing any therapy due to motivation deficits. Older patients (>77 years) and those with ≥7 lesions were particularly unsure why their conditions needed to be treated, although they carry a high risk of developing an invasive cSCC. As they were less motivated by treating physicians, relatives and third parties may be approached to assure adherence to treatment in this subgroup. Although the relatives’ desire for treatment was rated rather less important as motivation in the overall population, the values for this item were significantly higher for the subgroups age ≥77 years, men, and localization of lesions on the head or face.

Among treatment expectations, we identified two more clusters whose items were positively correlated. Patients of cluster 3 expected both efficacy and safety measures as well as no impairment in daily living along with the intervention. We conclude that patients of cluster 3 are therefore moderately motivated for treatment if everyday life is not affected by the interventions chosen. In contrast, safety, efficacy, and tolerability were the main domains for patients of cluster 4. Although these patients appear highly motivated to undergo treatment, they also have high expectations towards the interventions. We believe that considering and balancing these preferences will help to ensure adherence to treatment and facilitate ideal treatment outcomes.

We are aware that this study has several limitations. The sample comprised 403 patients recruited during a short period. This sample size is relatively small, and the study population was not sampled randomly but depending on the availability of patients. Most questionnaires have been obtained from the University Hospital Munich, hence this overrepresentation may skew the results, although we believe that geographic or inter-city diversity can be neglected due to the small size of Germany. Besides, participants with high cumulative sun exposure were underrepresented which may limit the external validity of this study. Thus, the results presented here may not be fully generalizable to the general population and are at risk for sampling bias.

## 5. Conclusions

This study provides a patient-based heuristic tool to facilitate personalized treatment decisions in patients with AK. Considering patient profiles and individual preferences are of paramount importance to ensure patient adherence and to achieve ideal treatment outcomes. Nevertheless, the choice of the intervention should be made on a case-by-case basis and thoroughly discussed to reach an informed treatment consensus.

## Figures and Tables

**Table 1:** Baseline characteristics of the study population.

Sample	% (*n*)
**Sex (*n* = 399)**	
Female	26.3 (105)
Male	73.7 (294)
**Age (*n* = 395)**	
years (median, range)	77 (43–94)
years (mean ± standard deviation)	75.10 ± 9.45
**Family status (*n* = 396)**	
Single/unmarried	5.3 (21)
Married	73.5 (291)
Divorced	5.6 (22)
Widowed	15.7 (62)
**Risk exposure for skin cancer (*n* = 333)**	
Yes	9.0 (30)
No	91.0 (303)
**Health insurance (*n* = 396)**	
Statutory health insurance	76.8 (304)
Private health insurance	23.2 (92)
**Immunosuppression (*n* = 382)**	
No	92.1 (352)
Yes	7.9 (30)
Organ transplant recipient	56.7 (17)
Autoimmune disease	16.7 (5)
Other	26.7 (8)
**Previous treatment of AK (*n* = 396)**	
Yes	83.6 (331)
No	15.4 (61)
Unsure	1.0 (4)
**Last treatment of AK (*n* = 292)**	
months (median, range)	6 (0–300)
months (mean ± standard deviation)	18.62 ± 32.35
**Number of AK to be treated at the visit (*n* = 351)**	
1–3	47.6 (167)
4–6	22.5 (79)
≥7	29.9 (105)
**Outdoor profession (*n* = 333)**	
Yes	9.0 (30)
No	91.0 (303)
**Localization of AK (*n* = 398)**	
Scalp	57.3 (228)
Face	61.6 (245)
Trunk	8.0 (32)
Extremities	31.4 (125)
Only face/scalp	65.5 (261)
Only trunk/extremities	10.6 (42)
All sites	23.9 (95)

**Table 2:** Summary of treatment motivation and treatment expectation profiles.

	Treatment Motivation			Treatment Expectation		
	Cluster 1	Cluster 2		Cluster 3		Cluster 4
**Leading item**	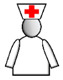 Physician recommendation	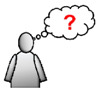 Unclear why AK need treatment		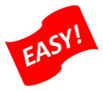 Simplicity	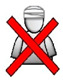 No impairment in daily life	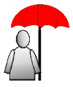 Safety
**Associated items**	(+) AK = precancerous condition Avoid transition to cSCC	(−) Physician recommendation AK = precancerous condition Avoid transition to cSCC	(+) Desire of relatives	(+) Safety Few adverse events Little painfulness	(+) Few adverse events Little painfulness	(+) Long-term efficacy Effective lesion clearance Few adverse events Little painfulness
**Patient characteristic**	Well informed about condition Fear of AK progress	Indifferent to condition May be approached by relatives Intrinsic and extrinsic motivation deficits		The convenient patient Motivated for treatment if it does not interfere with daily life		Discerning and rational-thinking patient Highly motivated but with high expectations of treatment
**Degree of motivation**	High	Low		Moderate		High
**Degree of expectation**	Moderate	Low		High		High

## Supplementary Materials

The following are available online at https://www.mdpi.com/2077-0383/9/5/1438/s1, Figure S1: Bar chart showing the distribution of patients’ previous interventions for AK in our sample; abbreviations: 5-FU = 5-fluorouracil; IMB = ingenol mebutate; PDT = photodynamic therapy; SA = salicylic acid, Supplementary A1: Ethical approval; Supplementary A2: STROBE checklist; Supplementary A3: Questionnaire regarding patients’ AK-specific motives and expectations distributed in German language.

## Appendix A

**Supplementary results: Subgroup analysis of treatment motivation items and subgroup analysis of treatment expectation items**

### Appendix A.1. Subgroup Analysis of Treatment Motivation Items

We revealed specific subgroup differences for gender and the willingness to undergo treatment due to the desire of relatives of the patient, i.e., men would rather receive treatment because their partners want them to do so (*p* = 0.017) (Figure 2A). The same motivation item also concerned patients aged ≥77 years in contrast to patients below that age (*p* = 0.004) and patients with AK located in the face or head region compared to trunk or extremities (*p* = 0.046). Additionally, older patients (≥77 years) were rather unsure about the necessity of treatment in comparison to younger patients (*p* = 0.001). Also, patients with ≥7 lesions were rather unsure about the necessity of treatment compared to those with 1–3 (*p* = 0.017) or 4–6 lesions (*p* = 0.016). Indoor workers stated that cosmesis was a motivation for treatment compared to outdoor workers (*p* = 0.039). Cosmetic outcome was also a pronounced motivation for immunocompetent patients compared to immunocompromised ones (*p* = 0.007). Notably, patients with statutory health insurance rather saw guideline recommendations as treatment motivation compared to those with private insurance (*p* = 0.000). Lastly, patients who have already undergone at least one prior treatment also considered the precancerous nature of AK as motivation (*p* = 0.005).

### Appendix A.2. Subgroup Analysis of Treatment Expectation Items

Women expected a good cosmetic result compared to men (*p* = 0.040) (Figure 2B). Patients with AK localized in the face or on the scalp also rather expected a good cosmetic outcome in comparison to those with AK on the extremities or trunk (*p* = 0.009). Furthermore, younger patients (<77 years) preferred to undergo treatment at home, whereas older patients preferred treatment at the hospital or practice (*p* = 0.006). Besides, treatment-naïve patients rather expected treatment to be performed only once (*p* = 0.001). Patients with AK located on the face or head area also desired treatment to be performed only once in comparison to those with AK located on every site of their body (*p* = 0.031). Additionally, patients with statutory health insurance preferred home-based treatment in contrast to those with private insurance (*p* = 0.001). Patients with statutory health insurance also expected low treatment costs (*p* = 0.000) and reimbursement by health insurance (*p* = 0.018) compared to private insurance. In comparison to single patients, married (*p* = 0.012) and widowed ones (*p* = 0.024) rather expected treatment to be hardly to little painful. Besides, patients with few AK (1–3) rather voted that treatment be carried out only once in comparison to those with 4–6 lesions (*p* = 0.025). However, in comparison to patients with a medium number of lesions (4–6), patients with 1–3 (*p* = 0.039) and patients with ≥7 lesions (*p* = 0.034) expected effective AK lesion clearance.

## References

[1] Salasche S.J. (2000). Epidemiology of actinic keratoses and squamous cell carcinoma. J. Am. Acad. Dermatol..

[2] Moy R.L. (2000). Clinical presentation of actinic keratoses and squamous cell carcinoma. J. Am. Acad. Dermatol..

[3] Röwert-Huber J., Patel M.J., Forschner T., Ulrich C., Eberle J., Kerl H., Sterry W., Stockfleth E. (2007). Actinic keratosis is an early in situ squamous cell carcinoma: A proposal for reclassification. Br. J. Dermatol..

[4] Criscione V.D., Weinstock M.A., Naylor M.F., Luque C., Eide M.J., Bingham S.F., for the Department of Veteran Affairs Topical Tretinoin Chemoprevention Trial Group (2009). Actinic keratoses: Natural history and risk of malignant transformation in the Veterans Affairs Topical Tretinoin Chemoprevention Trial. Cancer.

[5] Werner R.N., Sammain A., Erdmann R., Hartmann V., Stockfleth E., Nast A. (2013). The natural history of actinic keratosis: A systematic review. Br. J. Dermatol..

[6] Cerio R., Dirschka T., Dréno B., Nart I., Lear J., Pellacani G., Peris K., Casas A. (2017). Actinic Keratosis, a Chronic, Progressive Disease: Understanding Clinical Gaps to Optimise Patient Management. Acta Derm. Venereol..

[7] Schmitz L., Gambichler T., Kost C., Gupta G., Stucker M., Stockfleth E., Dirschka T. (2018). Cutaneous squamous cell carcinomas are associated with basal proliferating actinic keratoses. Br. J. Dermatol..

[8] Leiter U., Heppt M.V., Steeb T., Amaral T., Bauer A., Becker J.C., Breitbart E., Breuninger H., Diepgen T., Dirschka T. (2020). S3 guideline for actinic keratosis and cutaneous squamous cell carcinoma (cSCC)—Short version, part 2: Epidemiology, surgical and systemic treatment of cSCC, follow-up, prevention and occupational disease. J. Dtsch. Dermatol. Ges..

[9] Heppt M.V., Leiter U., Steeb T., Amaral T., Bauer A., Becker J.C., Breitbart E., Breuninger H., Diepgen T., Dirschka T. (2020). S3 guideline for actinic keratosis and cutaneous squamous cell carcinoma—Short version, part 1: Diagnosis, interventions for actinic keratoses, care structures and quality-of-care indicators. J. Dtsch. Dermatol. Ges..

[10] Dirschka T., Gupta G., Micali G., Stockfleth E., Basset-Séguin N., Del Marmol V., Dummer R., Jemec G., Malvehy J., Peris K. (2016). Real-world approach to actinic keratosis management: Practical treatment algorithm for office-based dermatology. J. Dermatol. Treat..

[11] Bridges J.F.P., Hauber B., Marshall D.A., Lloyd A., Prosser L.A., Regier D.A., Johnson F.R., Mauskopf J. (2011). Conjoint Analysis Applications in Health—A Checklist: A Report of the ISPOR Good Research Practices for Conjoint Analysis Task Force. Value Heath.

[12] Kopasker D., Kwiatkowski A., Matin R., Harwood C., Ismail F., Lear J., Thomson J., Hasan Z.-U., Wali G., Milligan A. (2018). Patient preferences for topical treatment of actinic keratoses: A discrete-choice experiment. Br. J. Dermatol..

[13] Cerio R. (2017). The importance of patient-centred care to overcome barriers in the management of actinic keratosis. J. Eur. Acad. Dermatol. Venereol..

[14] Berker D. (2019). A discrete-choice experiment and actinic keratosis: What is the answer?. Br. J. Dermatol..

[15] Reynolds K.A., Schlessinger D.I., Vasic J., Iyengar S., Qaseem Y., Behshad R., DeHoratius D.M., Denes P., Drucker A.M., Dzubow L.M. (2020). Core Outcome Set for Actinic Keratosis Clinical Trials. JAMA Dermatol..

[16] Von Elm E., Altman U.G., Egger M., Pocock S.J., Gøtzsche P.C., Vandenbroucke J.P. (2014). The Strengthening the Reporting of Observational Studies in Epidemiology (STROBE) Statement: Guidelines for reporting observational studies. Int. J. Surg..

[17] Vandenbroucke J.P., Von Elm E., Altman U.G., Gøtzsche P.C., Mulrow C.D., Pocock S.J., Poole C., Schlesselman J.J., Egger M. (2014). Strengthening the Reporting of Observational Studies in Epidemiology (STROBE): Explanation and elaboration. Int. J. Surg..

[18] Tabachniek B.G., Fidell L.S. (1984). Book Review: Reply to Widaman’s Review of Using Multivariate Statistics. Appl. Psychol. Meas..

[19] Philipp-Dormston W.G., Battistella M., Boussemart L., Di Stefani A., Broganelli P., Thoms K.-M. (2019). Patient-centered management of actinic keratosis. Results of a multi-center clinical consensus analyzing non-melanoma skin cancer patient profiles and field-treatment strategies. J. Dermatol. Treat..

[20] Maisel A., Waldman A., Furlan K., Weil A., Sacotte K., Lazaroff J.M., Lin K., Aranzazu D., Avram M.M., Bell A. (2018). Self-reported Patient Motivations for Seeking Cosmetic Procedures. JAMA Dermatol..

[21] Waldman A., Maisel A., Weil A., Iyengar S., Sacotte K., Lazaroff J.M., Kurumety S., Shaunfield S.L., Reynolds K.A., Poon E. (2019). Patients believe that cosmetic procedures affect their quality of life: An interview study of patient-reported motivations. J. Am. Acad. Dermatol..

[22] Storer M., Zhu Z., Sokil M., Ford M., Neugebauer R., Asgari M.M. (2017). Community-Based Practice Variations in Topical Treatment of Actinic Keratoses. JAMA Dermatol..

[23] Roman J., Elpern D.J. (2017). Helping Patients Decide on Treatment Options for Actinic Keratosis-Living in Cryo Nation. JAMA Dermatol..

[24] Noels E., Lugtenberg M., Egmond S., Droger S., Buis P., Nijsten T., Wakkee M. (2019). Insight into the management of actinic keratosis: A qualitative interview study among general practitioners and dermatologists. Br. J. Dermatol..

[25] Berry K., Butt M., Kirby J.S. (2017). Influence of Information Framing on Patient Decisions to Treat Actinic Keratosis. JAMA Dermatol..

