# Supplementary material for: Treatment Motivations and Expectations in Patients with Actinic Keratosis: A German-Wide Multicenter, Cross-Sectional Trial
Source: J Clin Med. 2020 May 12;9(5):1438. doi: 10.3390/jcm9051438 (PMC7290787; doi:10.3390/jcm9051438)
Supplement: Supplementary file 1 [file jcm-09-01438-s001.zip › jcm-777037-sm/Figure s1 .docx]

Supplementary Materials:


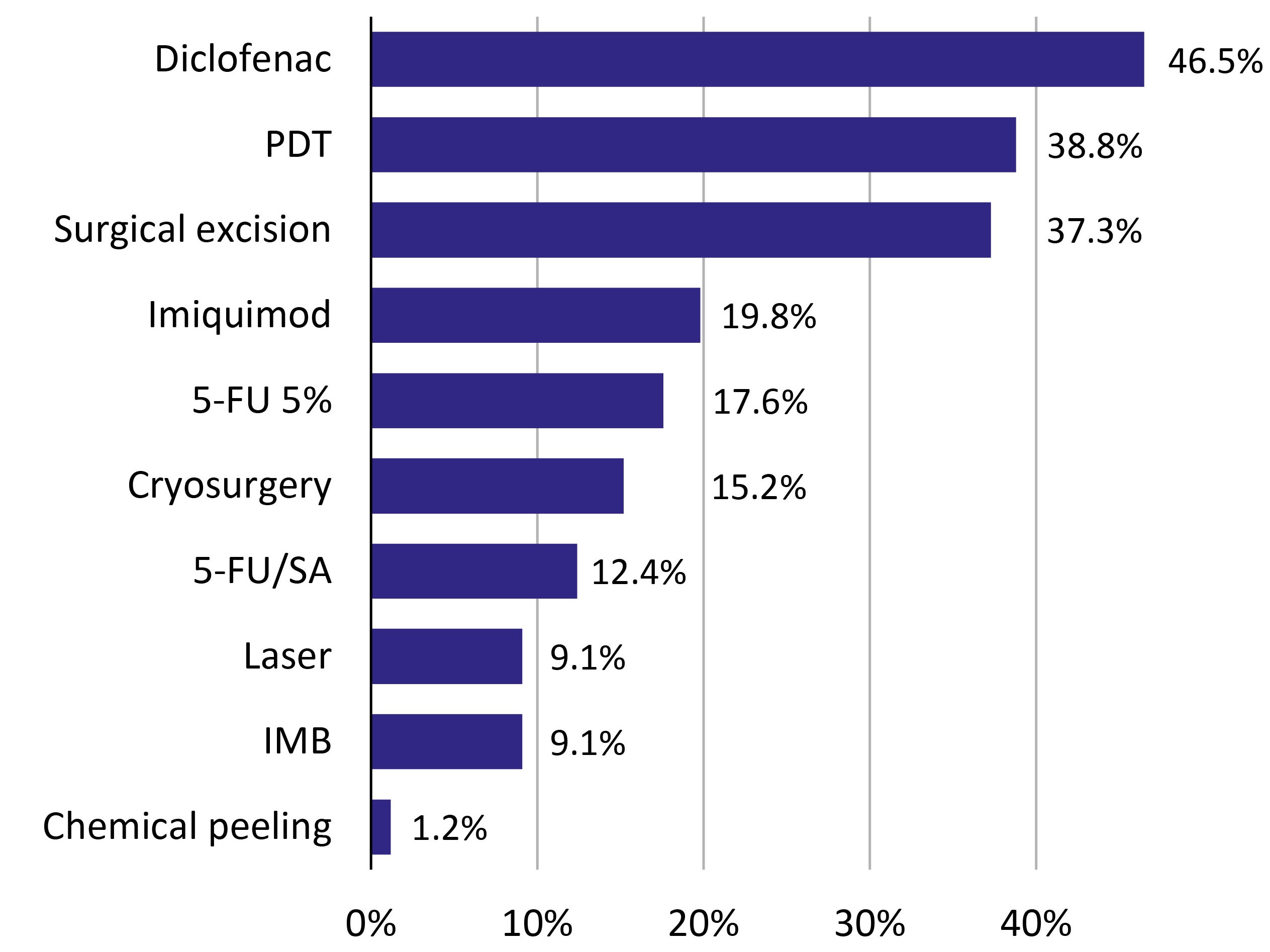


**Figure S1.** Bar chart showing the distribution of patients’ previous interventions for AK in our sample; abbreviations: 5-FU = 5-fluorouracil; IMB = ingenol mebutate; PDT = photodynamic therapy; SA = salicylic acid.
