# Supplementary material for: Treatment Motivations and Expectations in Patients with Actinic Keratosis: A German-Wide Multicenter, Cross-Sectional Trial
Source: J Clin Med. 2020 May 12;9(5):1438. doi: 10.3390/jcm9051438 (PMC7290787; doi:10.3390/jcm9051438)
Supplement: Supplementary file 1 [file jcm-09-01438-s001.zip › jcm-777037-sm/Suppl A1_ethical approval.pdf]

LMU LUDWIG-MAXIMILIANS-UNIVERSITÄT MÜNCHEN

ETHIKKOMMISSION BEI DER LMU MÜNCHEN

Ethikkommission ·Pettenkoferstr. 8 · 80336 München
Klinikum der Universität München
Klinik und Poliklinik für Dermatologie und Allergologie
Prof. Dr. Carola Berking
Frauenlobstr. 9 - 11
80337 München

Vorsitzender:
Prof. Dr. W. Eisenmenger
Telefon+49 (0)89 440055191
Telefax+49 (0)89 440055192
Ethikkommission@
med.uni-muenchen.de
www.ethikkommission.med.uni
-muenchen.de

Anschrift:
Pettenkoferstr. 8a
D-80336 München

Ansprechpartner:
Oskars Mikazans, M.A.

Telefon: 089-4400 57604
E-Mail: oskars.mikazans@
med.uni-muenchen.de

07.06.2019 Hb /om

Projekt Nr.: **19-356 KB** (bitte bei Schriftwechsel angeben)

Keine Beratungspflicht

Projekt:	Treatment motivation and expectation among patients with actinic keratosis: a cross-sectional survey
Antragsteller:	Prof. Dr. Carola Berking, Klinikum der Universität München, Klinik und Poliklinik für Dermatologie und Allergologie, Frauenlobstr. 9 - 11, 80337 München
Untersucher:	Prof. Dr. Carola Berking, Klinikum der Universität München, Klinik und Poliklinik für Dermatologie und Allergologie, Frauenlobstr. 9 - 11, 80337 München

Sehr geehrte Frau Prof. Berking,

besten Dank für Ihr Schreiben vom 04.05.2019, mit dem Sie um eine Stellungnahme zum o.g. Projekt bitten.

Da Sie Ihre Untersuchungen anhand von Datensätzen durchführen, die irreversibel anonymisiert sind, d. h. dass auch den Bearbeitern kein Rückschluss auf die personenbezogenen Daten der Probanden möglich ist, besteht keine Beratungspflicht durch die Ethikkommission.

Für Ihre Untersuchungen wünsche ich Ihnen viel Erfolg.

Mit freundlichen Grüßen

Prof. Dr. W. Eisenmenger
Vorsitzender der Ethikkommission

Mitglieder der Kommission:
Prof. Dr. W. Eisenmenger (Vorsitzender), Prof. Dr. E. Held (Vorsitzender), Prof. Dr. H. Angstwurm, Prof. Dr. S. Böck, J. Eckert, Prof. Dr. B. Emmerich, Prof. Dr. S. Endres, Prof. Dr. R. Fischer, Prof. Dr. H. U. Gallwas, Prof. Dr. O. Genzel- Boroviczény, Prof. Dr. K. Hahn, Prof. Dr. N. Harbeck, Dr. B. Henrikus, Prof. Dr. C. Heumann, Prof. Dr. A. Holstege, Prof. Dr. R. M. Huber, Prof. Dr. V. Klauss, Dr. F. Kohlmayer, Prof. Dr. J. Lindner, Prof. Dr. S. Lorenzl, Prof. Dr. G. Marckmann, Dr. V. Mönch, PD Dr. Dr. H. Mückter, Prof. Dr. A. Nassehi, Prof. Dr. R. Penning, Prof. Dr. J. Peters, Prof. Dr. K. Pfeifer, Dr. I. Saake, Prof. Dr. H. Schardey, Prof. Dr. M. Schmauss, Prof. Dr. U. Schroth, Prof. Dr. O. Steinlein, PD Dr. G. Stüben, Prof. Dr. H. Waldner, PD Dr. U. Wandl, Prof. Dr. C. Wendtner, Dr. A. Yassouridis, Dr. C. Zach
