# Supplementary material for: Treatment Motivations and Expectations in Patients with Actinic Keratosis: A German-Wide Multicenter, Cross-Sectional Trial
Source: J Clin Med. 2020 May 12;9(5):1438. doi: 10.3390/jcm9051438 (PMC7290787; doi:10.3390/jcm9051438)
Supplement: Supplementary file 1 [file jcm-09-01438-s001.zip › jcm-777037-sm/Suppl A2 & A3_Strobe and questionaire_NEW.docx]

**Supplementary A2**: STROBE Statement—Checklist of items that should be included in reports of ***cross-sectional studies***

**Profiling treatment motivations and expectations in patients with actinic keratosis: A German-wide multicenter, cross-sectional trial**

|  | Item No | Recommendation | Page No |
| --- | --- | --- | --- |
| **Title and abstract** | 1 | (*a*) Indicate the study’s design with a commonly used term in the title or the abstract | 1 |
|  |  | (*b*) Provide in the abstract an informative and balanced summary of what was done and what was found | 2 |
| Introduction | | | |
| Background/rationale | 2 | Explain the scientific background and rationale for the investigation being reported | 2 |
| Objectives | 3 | State specific objectives, including any prespecified hypotheses | 2-3 |
| Methods | | | |
| Study design | 4 | Present key elements of study design early in the paper | 3 |
| Setting | 5 | Describe the setting, locations, and relevant dates, including periods of recruitment, exposure, follow-up, and data collection | 3 |
| Participants | 6 | (*a*) Give the eligibility criteria, and the sources and methods of selection of participants | 3 |
| Variables | 7 | Clearly define all outcomes, exposures, predictors, potential confounders, and effect modifiers. Give diagnostic criteria, if applicable | 3 |
| Data sources/ measurement | 8* | For each variable of interest, give sources of data and details of methods of assessment (measurement). Describe comparability of assessment methods if there is more than one group | *3* |
| Bias | 9 | Describe any efforts to address potential sources of bias | 3 |
| Study size | 10 | Explain how the study size was arrived at | 3 |
| Quantitative variables | 11 | Explain how quantitative variables were handled in the analyses. If applicable, describe which groupings were chosen and why | 3-4 |
| Statistical methods | 12 | (*a*) Describe all statistical methods, including those used to control for confounding | 3-4 |
|  |  | (*b*) Describe any methods used to examine subgroups and interactions | 3-4 |
|  |  | (*c*) Explain how missing data were addressed | 4 |
|  |  | (*d*) If applicable, describe analytical methods taking account of sampling strategy | 3-4 |
|  |  | (*e*) Describe any sensitivity analyses | n.a. |
| Results | | | |
| Participants | 13* | (a) Report numbers of individuals at each stage of study—eg numbers potentially eligible, examined for eligibility, confirmed eligible, included in the study, completing follow-up, and analysed | 4 |
|  |  | (b) Give reasons for non-participation at each stage | n.a. |
|  |  | (c) Consider use of a flow diagram | n.a. |
| Descriptive data | 14* | (a) Give characteristics of study participants (eg demographic, clinical, social) and information on exposures and potential confounders | Table 1, page 4-12 |
|  |  | (b) Indicate number of participants with missing data for each variable of interest | Table 1, Figures, 4-12 |
| Outcome data | 15* | Report numbers of outcome events or summary measures | 4 |
| Main results | 16 | (*a*) Give unadjusted estimates and, if applicable, confounder-adjusted estimates and their precision (eg, 95% confidence interval). Make clear which confounders were adjusted for and why they were included | 4-12 |
|  |  | (*b*) Report category boundaries when continuous variables were categorized | figures |
|  |  | (*c*) If relevant, consider translating estimates of relative risk into absolute risk for a meaningful time period | n.a. |
| Other analyses | 17 | Report other analyses done—eg analyses of subgroups and interactions, and sensitivity analyses | supplementary |
| Discussion | | | |
| Key results | 18 | Summarise key results with reference to study objectives | 12-14 |
| Limitations | 19 | Discuss limitations of the study, taking into account sources of potential bias or imprecision. Discuss both direction and magnitude of any potential bias | 14 |
| Interpretation | 20 | Give a cautious overall interpretation of results considering objectives, limitations, multiplicity of analyses, results from similar studies, and other relevant evidence | 14 |
| Generalisability | 21 | Discuss the generalisability (external validity) of the study results | 14 |
| Other information | | | |
| Funding | 22 | Give the source of funding and the role of the funders for the present study and, if applicable, for the original study on which the present article is based | 14 |

*Give information separately for exposed and unexposed groups.

**Note:** An Explanation and Elaboration article discusses each checklist item and gives methodological background and published examples of transparent reporting. The STROBE checklist is best used in conjunction with this article (freely available on the Web sites of PLoS Medicine at http://www.plosmedicine.org/, Annals of Internal Medicine at http://www.annals.org/, and Epidemiology at http://www.epidem.com/). Information on the STROBE Initiative is available at [www.strobe-statement.org](http://www.strobe-statement.org).

**Supplementary A3**: Befragung von Patienten mit aktinischen Keratosen zur Motivation und Erwartung an die Therapie

Sehr geehrte Damen und Herren,

zunächst möchten wir Ihnen für Ihre Bereitschaft danken, an dieser Befragung teilzunehmen. Im folgenden Fragebogen werden Angaben zu Ihrer Motivation und Erwartung zur Behandlung aktinischer Keratosen erhoben.

Die Beantwortung des Fragebogens ist freiwillig und wird nur wenige Minuten in Anspruch nehmen. Lesen Sie sich die Fragen bitte aufmerksam durch und kreuzen Sie die für Sie zutreffenden Antworten an. Der Fragebogen umfasst insgesamt 4 Seiten.

Bitte füllen Sie Frage 1 bis 4 auf Seite 1 gegebenenfalls zusammen mit Ihrem behandelnden Arzt aus; die restlichen Fragen füllen Sie bitte selbst aus.

Sollten Sie Fragen haben, können Sie sich über die onkologische Ambulanz an Herrn Dr. Markus Heppt wenden. Ihre Angaben werden selbstverständlich vertraulich behandelt und lediglich in anonymisierter Form verarbeitet.

Mit freundlichen Grüßen


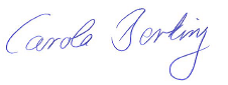


Prof. Dr. med. Carola Berking

Oberärztin

Onkologische Ambulanz

Klinik und Poliklinik für Dermatologie und Allergologie

Klinikum der Universität München
Campus Innenstadt

Frauenlobstr. 9-11

80337 München

Studienarzt:

Dr. med. Markus Heppt

🖂 Markus.Heppt@med.uni-muenchen.de

**Bitte füllen Sie Frage 1 bis 5 auf Seite 1 gegebenenfalls zusammen mit Ihrem Arzt aus!**

**Frage 1a: Wurden Ihre aktinischen Keratosen bereits in der Vergangenheit behandelt?**

🞏 Ja 🡪 weiter bei Frage 1b 🞏 Nein 🡪 weiter bei Frage 2 🞏 weiß nicht🡪 weiter bei Frage 2

**Frage 1b: Falls ja: Wie wurden Ihre aktinischen Keratosen bereits behandelt? (Mehrfachantworten möglich)**

🞏 Photodynamische Therapie

🞏 konventionelle photodynamische Therapie mit LED

🞏 Photodynamische Therapie mit Tageslicht

🞏 Diclofenac-Natrium 3% in 2.5% Hyaluronsäure-Gel (z.B. Solaraze®,

Solacutan®)

🞏 5% 5-Fluorouracil Creme (Efudix®)

🞏 5-Fluorouracil mit Salicylsäure (Actikerall®)

🞏 Ingenolmebutat Gel (Picato®)

🞏 Imiquimod Creme (z.B. Zyclara®, Aldara®)

🞏 Lasertherapie

🞏 Chirurgische Entfernung

🞏 Kryochirugie (=Vereisung)

🞏 Sonstige Therapie

Bitte um Angabe der Behandlung:

**Frage 1c: Wann fand die letzte Behandlung Ihrer aktinischen Keratosen statt? (Angabe bitte in Jahren ODER Monaten)**

Vor ____ Jahren bzw. vor ____ Monaten

**Frage 2: Wie viele aktinische Keratosen sollen heute behandelt werden?**

🞏 1 – 3 🞏 4 – 6 🞏 ≥7

**Frage 3: Wo befinden sich Ihre aktinischen Keratosen (Mehrfachantworten möglich)**

🞏 Kopfhaut 🞏 Körperstamm

🞏 Gesicht 🞏 Arm(e) und/oder Bein(e)

**Frage 4a: Nehmen Sie regelmäßig immunsupprimierende Medikamente ein?**

🞏 Nein 🡪 Weiter bei Frage 5 auf Seite 2

🞏 Ja 🡪 Weiter bei Frage 4b

**Frage 4b: Aus welchem Grund nehmen Sie immunsupprimierende/immunschwächende Medikamente ein?**

🞏 Organtransplantation

Bitte um Angabe des transplantierten Organs:

🞏 Autoimmunerkrankung

🞏 Weitere Gründe:

**Frage 5: In dem untenstehenden Block haben wir eine Reihe von Aussagen zu *Ihrer Motivation zu einer Therapie* Ihrer aktinischen Keratosen aufgeführt.**

**Bitte lesen Sie jede Aussage und kreuzen Sie auf der Linie an, inwieweit Sie dieser zustimmen oder nicht zustimmen.**

Stimme gar nicht zu

Stimme voll und ganz zu

Unentschieden

| Ich lasse meine aktinischen Keratosen aus **kosmetischen** Gründen behandeln.  Stimme gar nicht zu  Stimme voll und ganz zu  Unentschieden |  |
| --- | --- |
| Ich lasse meine aktinischen Keratosen behandeln, da der **Arzt** mir eine Therapie empfohlen hat.  Stimme gar nicht zu  Stimme voll und ganz zu  Unentschieden |  |
| Ich lasse meine aktinischen Keratosen behandeln, da diese als **Krebsvorstufe** angesehen werden.  Stimme gar nicht zu  Stimme voll und ganz zu  Unentschieden |  |
| Ich lasse meine aktinischen Keratosen behandeln, um eine **Weiterentwicklung in invasiven Hautkrebs** zu vermeiden.  Stimme gar nicht zu  Stimme voll und ganz zu  Unentschieden |  |
| Ich lasse meine aktinischen Keratosen behandeln, weil es in **Behandlungsrichtlinien** (medizinischen Leitlinien) empfohlen wird.  Stimme gar nicht zu  Stimme voll und ganz zu  Unentschieden |  |
| Ich lasse meine aktinischen Keratosen behandeln, da meine **Angehörigen** eine Therapie wünschen.  Stimme gar nicht zu  Stimme voll und ganz zu  Unentschieden |  |
| Der Grund, weshalb meine aktinischen Keratosen behandelt werden sollen, ist mir **unklar**.  Stimme gar nicht zu  Stimme voll und ganz zu  Unentschieden |  |

| Haben Sie noch **weitere Gründe oder Motivation** für eine Behandlung Ihrer aktinischen Keratosen, die nicht in der Tabelle aufgeführt sind?  **Weitere Gründe:** |
| --- |

**Frage 6: In dem untenstehenden Block haben wir eine Reihe von Aussagen zu einer möglichen Erwartung an die Therapie Ihrer aktinischen Keratosen aufgeführt.**

**Bitte lesen Sie jede Aussage und kreuzen Sie auf der Linie an, inwieweit Sie dieser zustimmen oder nicht zustimmen.**

| Bei einer Behandlung von aktinischen Keratosen ist für **mich** wichtig: | |
| --- | --- |
| Stimme gar nicht zu  Stimme voll und ganz zu  Unentschieden  Behandlungsdauer ist **kurz**. |  |
| Stimme gar nicht zu  Stimme voll und ganz zu  Unentschieden  Behandlung erfolgt **einmalig**. |  |
| Stimme gar nicht zu  Stimme voll und ganz zu  Unentschieden  Behandlung kann **zuhause** erfolgen. |  |
| Stimme gar nicht zu  Stimme voll und ganz zu  Unentschieden  Behandlung führt zu einer **effektiven Abheilung** der betroffenen Läsionen**.** |  |
| Stimme gar nicht zu  Stimme voll und ganz zu  Unentschieden  Behandlung hat eine nachgewiesene **Langzeitwirkung**. |  |
| Stimme gar nicht zu  Stimme voll und ganz zu  Unentschieden  Behandlung ist **sicher**. |  |
| Stimme gar nicht zu  Stimme voll und ganz zu  Unentschieden  Behandlung ist **einfach**. |  |
| Stimme gar nicht zu  Stimme voll und ganz zu  Unentschieden  Behandlung hat keine bis wenig **Nebenwirkungen**. |  |
| Behandlung erzielt ein gutes **kosmetisches** Ergebnis.  Stimme gar nicht zu  Stimme voll und ganz zu  Unentschieden |  |
| Stimme gar nicht zu  Stimme voll und ganz zu  Unentschieden  Die **Kosten** der Behandlung sind niedrig. |  |

| Bei einer Behandlung von aktinischen Keratosen ist für **mich** wichtig: | |
| --- | --- |
| Stimme gar nicht zu  Stimme voll und ganz zu  Unentschieden  Behandlung wird durch die **Krankenversicherung** erstattet. |  |
| Stimme gar nicht zu  Stimme voll und ganz zu  Unentschieden  Behandlung ist kaum bis wenig **schmerzhaft**. |  |
| Behandlung **beeinträchtigt** mich nicht im Alltag.  Stimme gar nicht zu  Stimme voll und ganz zu  Unentschieden |  |

| Haben Sie noch **weitere Erwartungen an die Behandlung** Ihrer aktinischen Keratosen, die nicht in der Tabelle aufgeführt sind?  **Weitere Erwartungen**: |
| --- |

**Zum Schluss noch einige Angaben zu Ihrer Person:**

**Sind Sie männlich oder weiblich?**

🞏 Männlich 🞏 Weiblich

**Bitte geben Sie Ihr Alter an:**

🞏 Jahre

**Familienstand**

🞏 ledig

🞏 verheiratet

🞏 geschieden

🞏 verwitwet

**In welchem Beruf haben Sie die längste Zeit gearbeitet?**

**Wie sind Sie aktuell krankenversichert?**

🞏 gesetzlich versichert

🞏 privat versichert

🞏 keine Versicherung

**Sie sind nun am Ende des Fragebogens angelangt.** **Vielen Dank für Ihre Teilnahme.**
